# Supplementary material for: Coupling nitrogen removal and watershed management to improve global lake water quality
Source: Nat Commun. 2025 Mar 4;16:2182. doi: 10.1038/s41467-025-57442-0 (PMC11880403; doi:10.1038/s41467-025-57442-0)
Supplement: Supplementary file 1 — Supplementary information [file 41467_2025_57442_MOESM1_ESM.pdf]

Supplementary information for

Coupling nitrogen removal and watershed management to improve global lake water quality

Authors:

Xing Yan[1,#], Yongqiu Xia[1,2,#,*], Xu Zhao[1,2], Chaopu Ti[1], Longlong Xia[1], Scott X. Chang[3], Xiaoyuan Yan[1,2,*]

Affiliations:

[1] State Key Laboratory of Soil and Sustainable Agriculture, Changshu National Agro-Ecosystem Observation and Research Station, Institute of Soil Science, Chinese Academy of Sciences, Nanjing, 211135, PR China

[2] University of Chinese Academy of Sciences, Nanjing, 211135, PR China

[3] Department of Renewable Resources, University of Alberta, Edmonton, Alberta, T6G 2R3, Canada

[#]These authors contributed equally to this paper: Xing Yan, Yongqiu Xia

[*]To whom correspondence should be addressed

E-mail: yqxia@issas.ac.cn, yanxy@issas.ac.cn

Supplementary Note 1

Model performance comparison between our remote sensing model and the RivR-N model for global lake N removal estimation

By comparing the estimated global lake N removals from our remote sensing model with the results of the RivR-N model[1], we validated our remote sensing model. As shown in Supplementary Fig. 10, the linear fitting of the blue line suggests that our estimations fit well with the results of the RivR-N model (R^2 = 0.48, P < 0.01). Moreover, the slope of the linear fitting is closely aligned with 1 (0.99 in Supplementary Fig. 10), demonstrating that our remote sensing model is generally consistent with the RivR-N model. However, Supplementary Fig. 10 also shows a discrepancy between our remote sensing model and the RivR-N model (the green line). This difference is likely due to our model incorporating the influence of substrate concentration on lake N removal rate. In fact, following the Michaelis-Menten equation, as substrate N concentration increases, lake N removal rate (or mass) slowly increases and approaches saturation due to limitations in enzyme activity (Supplementary Fig. 11). This phenomenon, known as the biological saturation effect under high N levels, is widely documented in the literature[2,3]. Consequently, the biological saturation effect leads to a decreasing lake N removal ratio (calculated by dividing the N removal rate by the substrate N loading) with the increasing substrate concentration. However, the RivR-N model, which considers the effect of water residence time and depth on lake N removal while focusing on physical effects, may fail to account for this biological saturation

effect. Hence, our remote sensing model, incorporating the effect of substrate concentration on lake N removal, generally provides a lower estimation of lake N removal compared to the RivR-N model.

47 **Supplementary Table 1 Aquatic ecosystem denitrification N removal estimated by environmental factors.**

Factors	R^2/r	n	Aquatic ecosystem	Study region	Reference
Water NO_3^- and, water temperature	$R^2 = 0.86$, $P = 0.000$	18	River, pond, and reservoir	Jurong reservoir watershed, eastern China	Li et al.[4]
Water NO_3^- and water temperature	$R^2 = 0.85$, $P = 0.000$	*36*	River	Lake Taihu basin	Zhao et al.[5]
Water NO_3^- and water temperature	$R^2 = 0.78$, $P = 0.000$	21	River	Lake Taihu basin	Zhao et al.[5]
Water NO_3^-	$R^2 = 0.86$, $P = 0.000$	136	Oceans, estuaries, lakes, and rivers	A global meta-analysis	Piña-Ochoa and Álvarez-Cobelas[6]
Water NO_3^-	$R^2 = 0.53$	23	Lake	Lake Shelbyville, Mississippi River basin	David et al.[7]
Water Chl*a*	$R^2 = 0.52$, $P = 0.000$	30	Lake	New Zealand	Bruesewitz et al.[8]
Water TN	$R^2 = 0.51$, $P = 0.000$	30	Lake	New Zealand	Bruesewitz et al.[8]
Water NO_3^-	$r = 0.51$, $P < 0.05$	15	Lake	Lake Bosten (China)	Jiang et al.[9]

Water NO_3^-	$R^2 = 0.87$, $P < 0.05$	21	Lake	Swiss	Müller et al.[10]
Water NO_3^-	$r = 0.66$, $P < 0.05$	10	Lake	Yangtze River basin	Liu et al.[11]
Water NO_3^- and water temperature	$R^2 = 0.48$, $P < 0.05$	11	Lake	Pyrenees	Palacin-Lizarbe et al.[12]
Water NO_3^-	$r = 0.53$, $P < 0.01$	24	Lake	Meiliang Bay and Inner Bay, Lake Taihu basin	Zhong et al.[13]
Water DIN and water temperature	$R^2 = 0.58$, $P < 0.01$	20	Drainage ditches	Zhushanwan watershed, Lake Taihu basin	She et al.[14]
Water NO_3^-	$r = 0.61$, $P < 0.01$	48	Lake	Lake Taihu	Liu et al.[11]
Water NO_3^-	$r = 0.78$, $P < 0.01$	75	Lake	Poyang Lake	Yao et al.[15]
Water NO_3^-	$r = 0.79$, $P < 0.01$	90	Lake	Poyang Lake	Zhang et al.[16]
Water DIN	$R^2 = 0.76$	13	River	Jiulong River, southeast China	Chen et al.[17]
Water NO_3^-	$r = 0.45$, $P < 0.01$	16	Ponds, ditches, and streams	Dongting Lake basin	Yan et al.[18]

Supplementary Table 2 Distribution of lake trophic state in this study.

	Oligotrophic	Mesotrophic	Eutrophic	Hypereutrophic
	Chl*a* <2 μg L^{-1}	2≤ Chl*a* <7 μg L^{-1}	7≤ Chl*a* < 30 μg L^{-1}	Chl*a* ≥30 μg L^{-1}
Lake number count	474	2,701	1,862	731
Trophic state distribution (%)	8.22	46.83	32.28	12.67

Supplementary Table 3 Lake counts requiring different times to achieve the water quality goal (1 mg N L^{-1}) under different watershed N input reduction scenarios.

Watershed N input reduction scenarios (% reduction)	Number of lakes that can achieve the water quality goal				
	0–10 yr	10–20 yr	20–30 yr	30–40 yr	>40 yr
Low-level (2%)	315	114	46	20	39
Intermediate-level (5%)	393	82	22	13	24
High-level (10%)	438	54	14	9	19

Supplementary Table 4 Watershed net anthropogenic N input (NANI) reduction to achieve the lake water quality goal (1 mg N L^{-1}) within 10, 20, 30, and 40 years for different regions.

Region	Watershed N input reduction (kg N km^{-2} yr^{-1})			
	10 yr	20 yr	30 yr	40 yr
North America	70.3	24.5	12.8	8.0
Europe	135.7	62.2	37.1	26.9
Asia	538.1	134.2	84.6	49.2

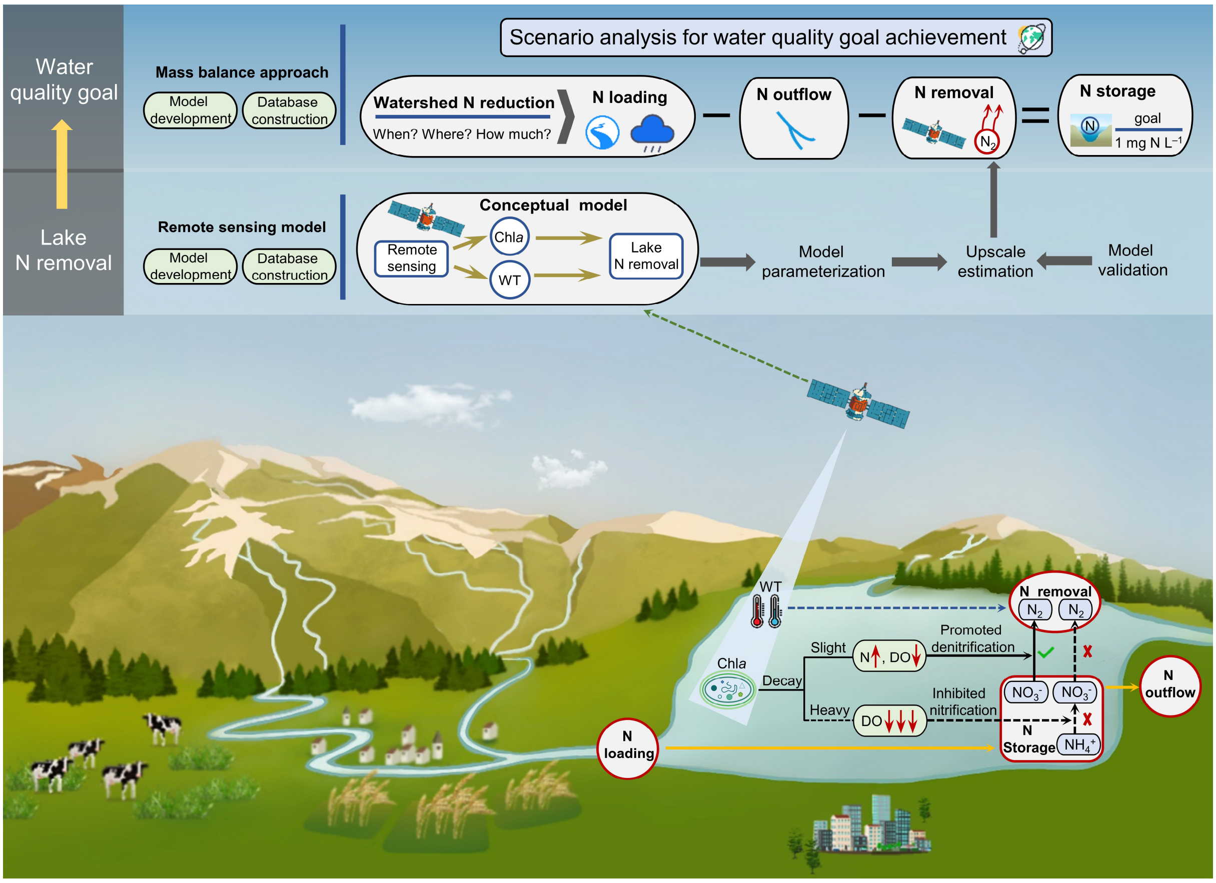

Supplementary Fig. 1 The development of the mass balance approach for the scenario analysis of water quality goal achievement based on the main lake N budget and lake N removal. The main N budget for the lake includes the N loading through surrounding rivers and underground loads and direct atmospheric N deposition to the lake, permanent N removal through denitrification, N storage in the lake, and N output through downstream rivers outflow.

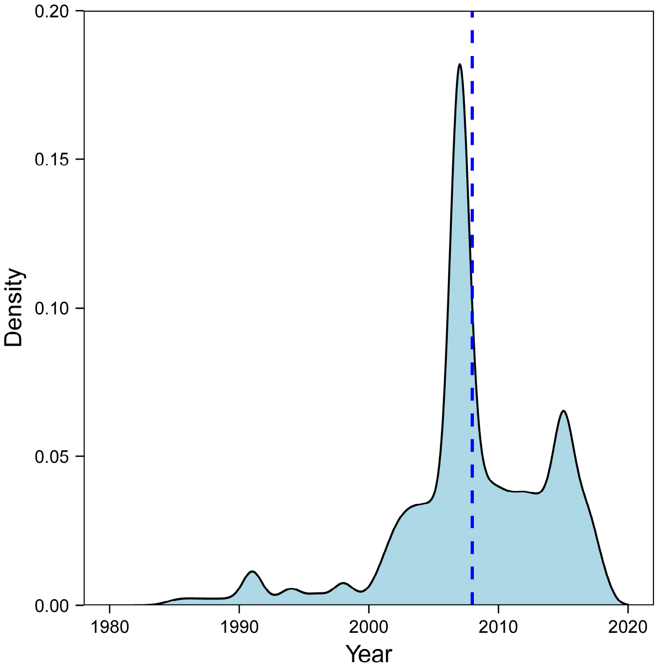

Supplementary Fig. 2 The probability distribution of the year for the interaction of the Chlorophyll-*a* and Water Chemistry datasets[20]. The colored vertical lines represent the mean values.

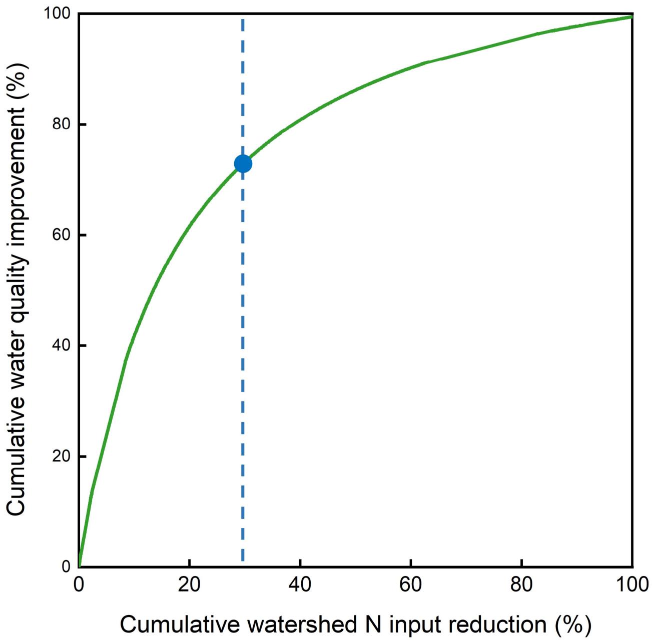

Supplementary Fig. 3 Relationship between cumulative water quality improvement and cumulative watershed N input reduction in this study. The dotted line shows that more than 70% of the cumulative water quality improvement could be achieved through a 30% reduction in the cumulative N loading.

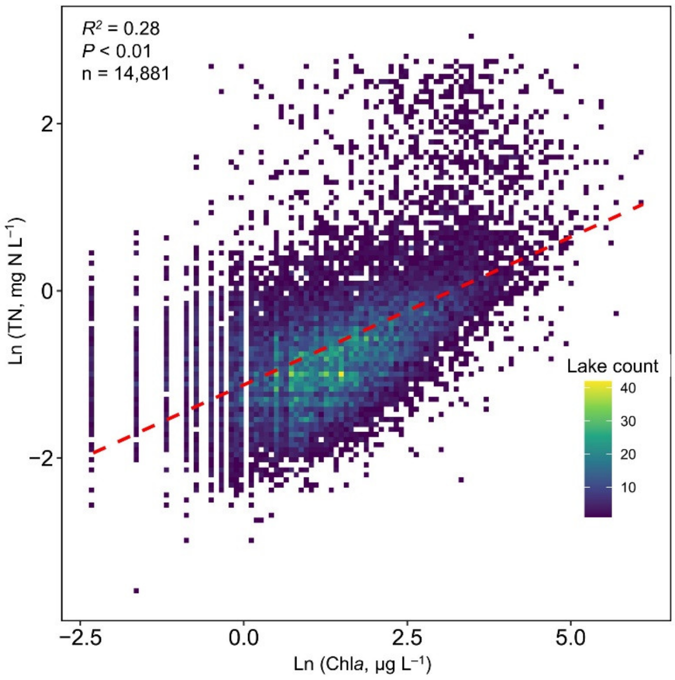

Supplementary Fig. 4 The relationship between Chl*a* and TN concentrations for 14,881 P-limited lakes with N:P ratio >22.4. Data were synthesized from the same dataset as those in this study[19].

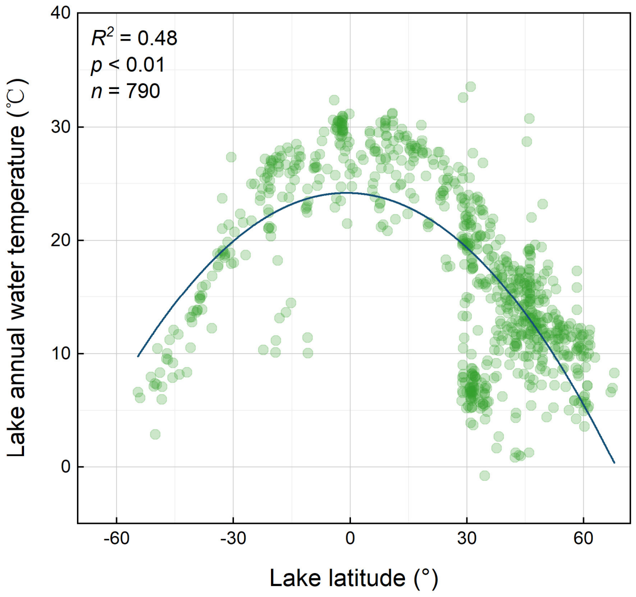

Supplementary Fig. 5 The relationship between global lake annual surface water temperature and lake latitude. Data were synthesized from the Copernicus Global Land Service database[20].

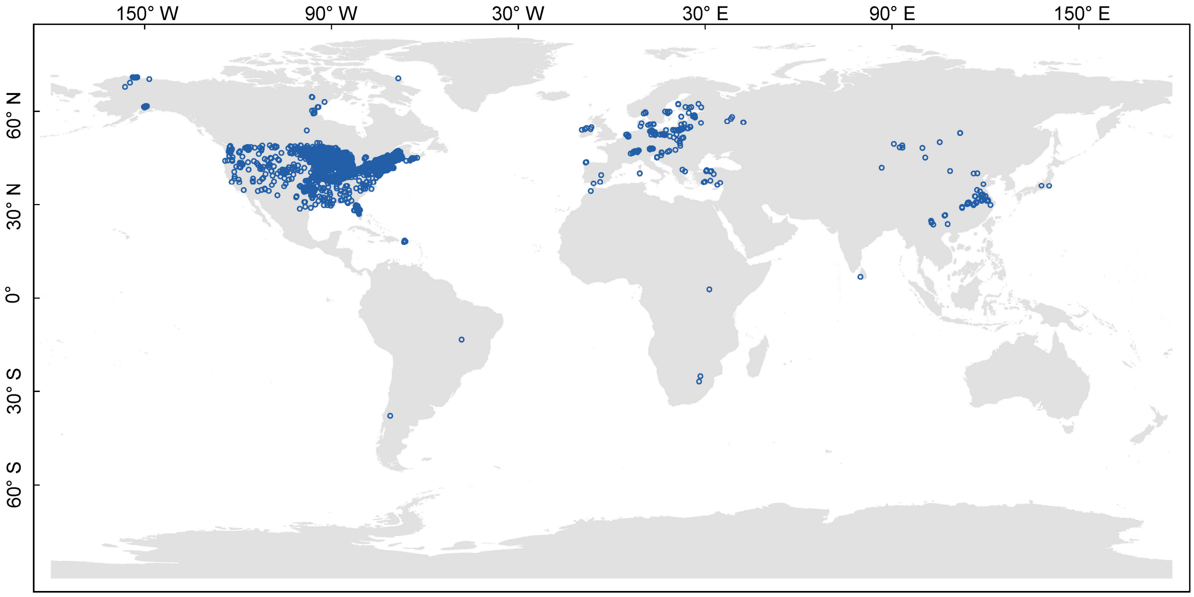

Supplementary Fig. 6 The spatial distribution of lakes analyzed in this study. A total of 5,768 lakes are identified based on the interaction of the Chlorophyll-*a* and Water Chemistry datasets and the HydroLAKES database. These lakes mainly include 5,515 lakes in North America, 187 lakes in Europe, and 59 lakes in Asia.

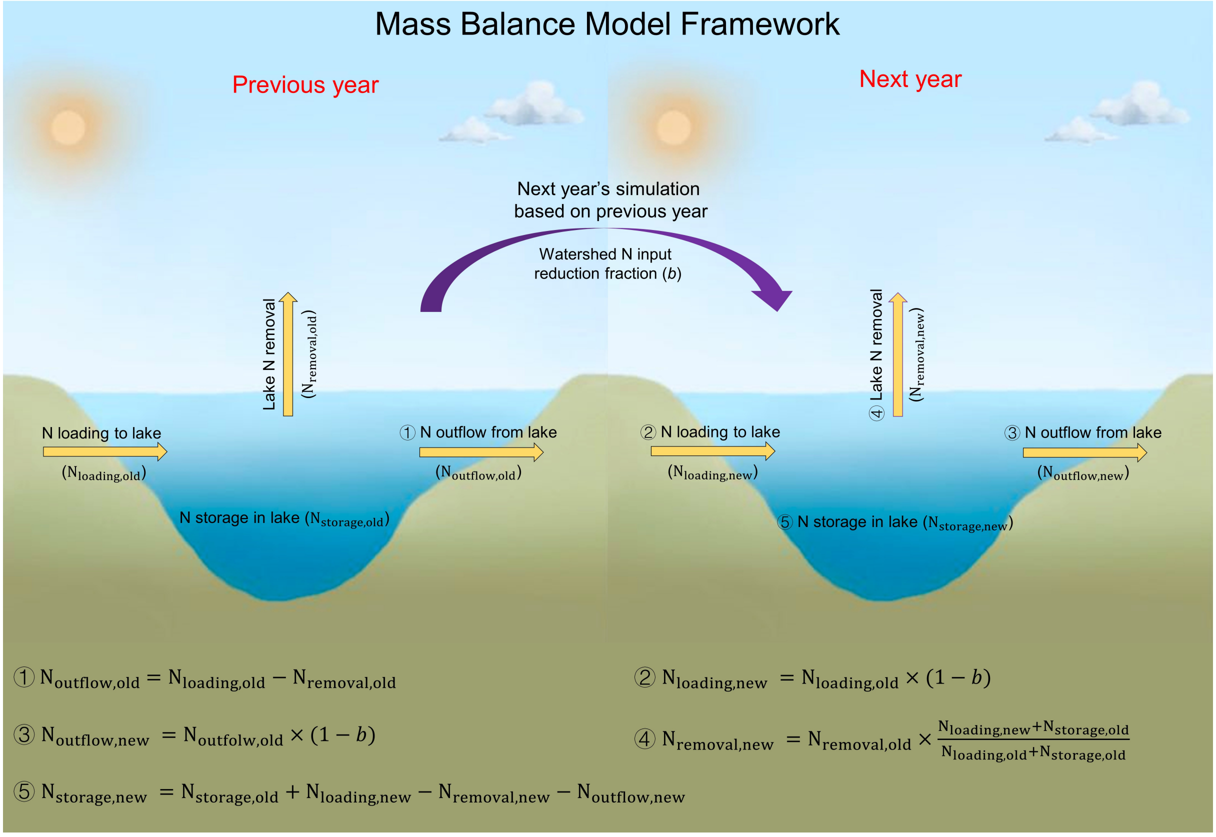

Supplementary Fig. 7 Mass balance model to simulate how lake water quality improves with watershed N management. The main N budget of the lake includes N loading to the lake ($N_{loading}$), N removed through N_2 emission ($N_{removal}$), N output through downstream rivers outflow ($N_{outflow}$), and the current lake N storage ($N_{storage}$). We use this as an example to illustrate how to simulate the lake N budget in the following year based on the lake N budget in each preceding year under watershed N management.

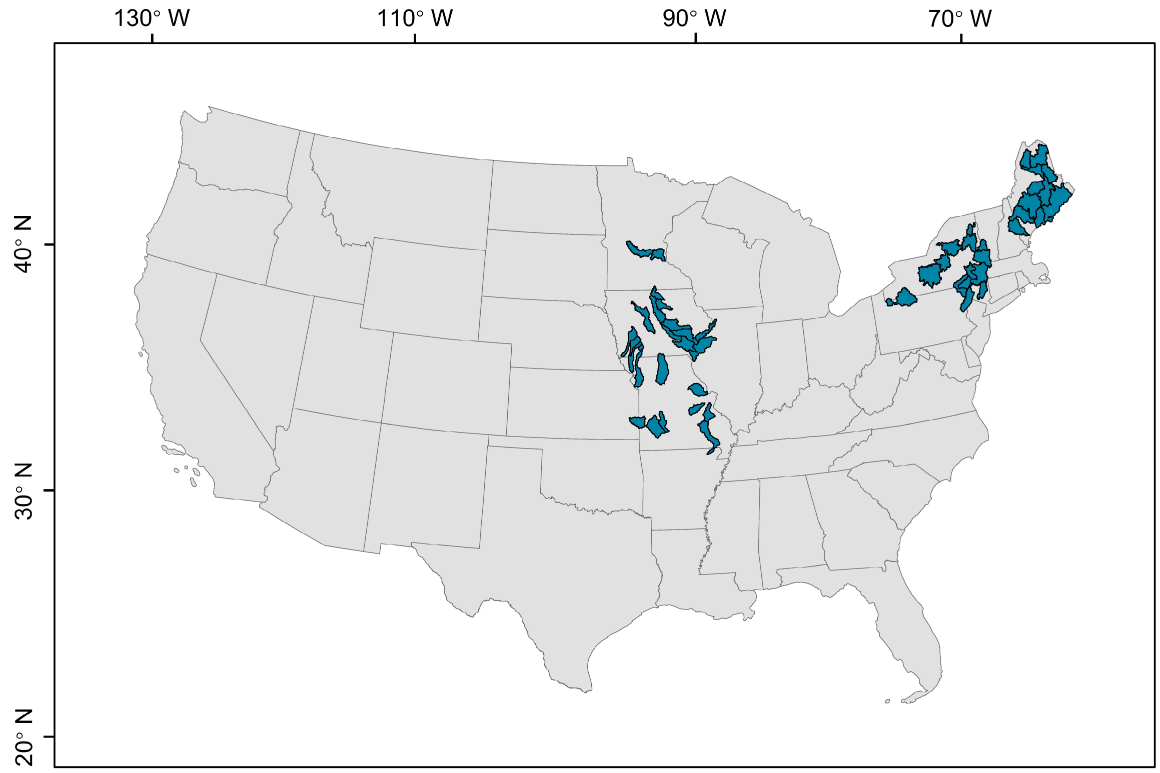

Supplementary Fig. 8 Spatial distributions of the 49 HUC-8 watersheds in northeastern US for validating the mass balance model. The blue polygons represent the HUC-8 watersheds used for validation.

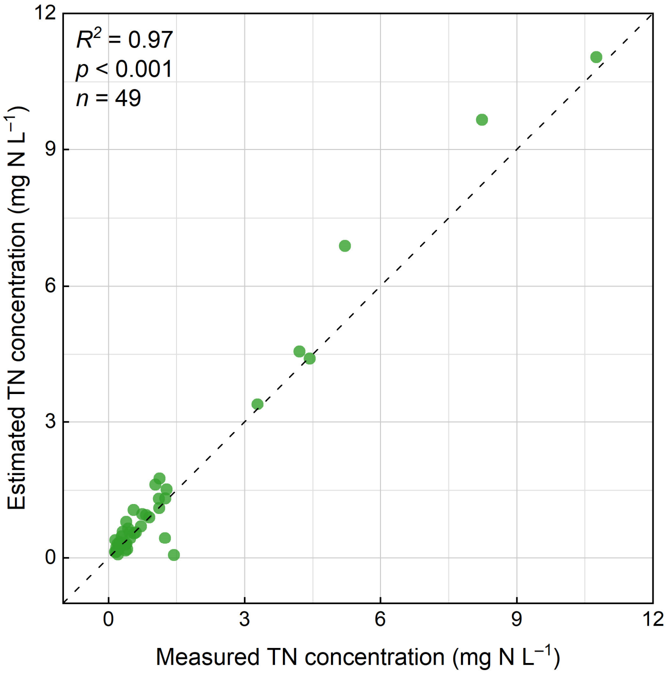

Supplementary Fig. 9 The validation of the mass balance model to simulate the response of water quality improvement to 10 years of watershed N management. The figure shows the relationship between estimated TN concentrations using the mass balance model and measured TN concentration in 49 HUC-8 watersheds of the northeastern US. Each point represents a HUC-8 watershed.

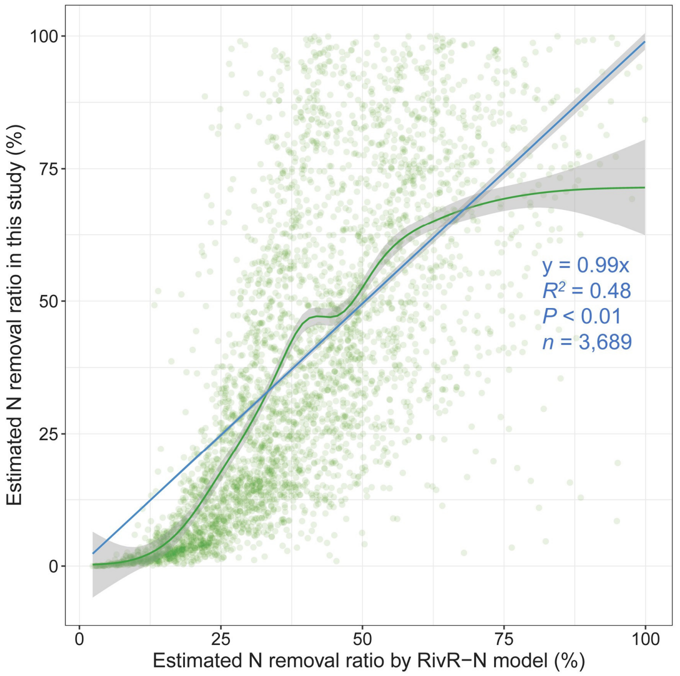

Supplementary Fig. 10 The relationship between the estimated global lake N removal by the remote sensing model established in this study and the RivR-N model. The RivR-N model was empirically derived from water residence time and depth for lake N removal ratio estimations across America and Europe[1].

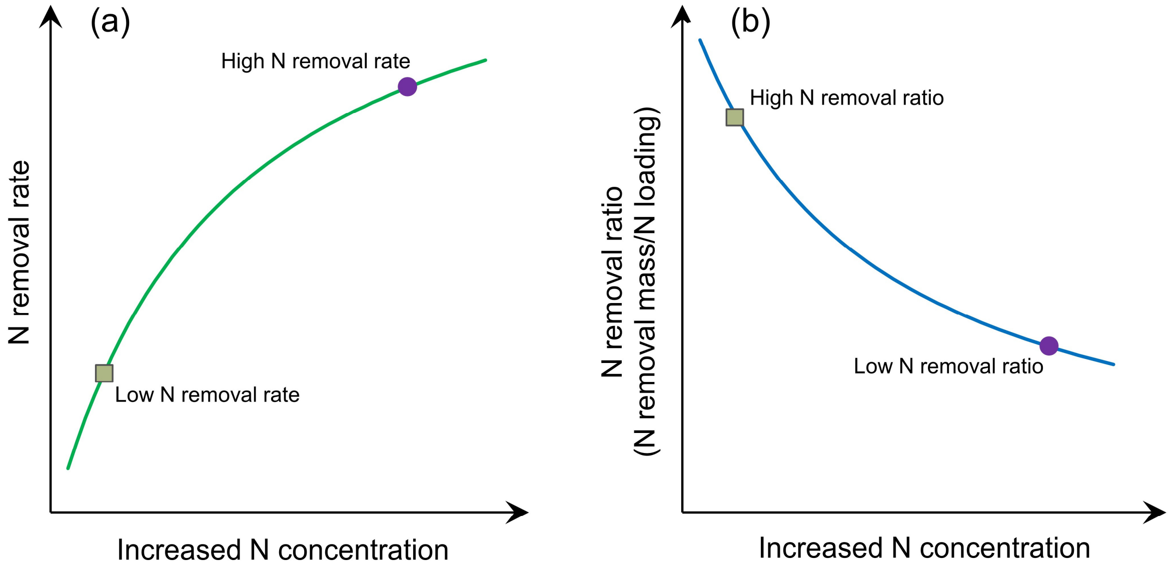

Supplementary Fig. 11 (a) relationship between increased substrate N concentration and N removal rate within the Michaelis-Menten equation, and (b) relationship between increased substrate N concentration and N removal ratio (characterized by N removal mass divided by substrate N loading) within the Michaelis-Menten equation. The squares and triangles represent the different N removal rates and ratios under the biological saturation effect.

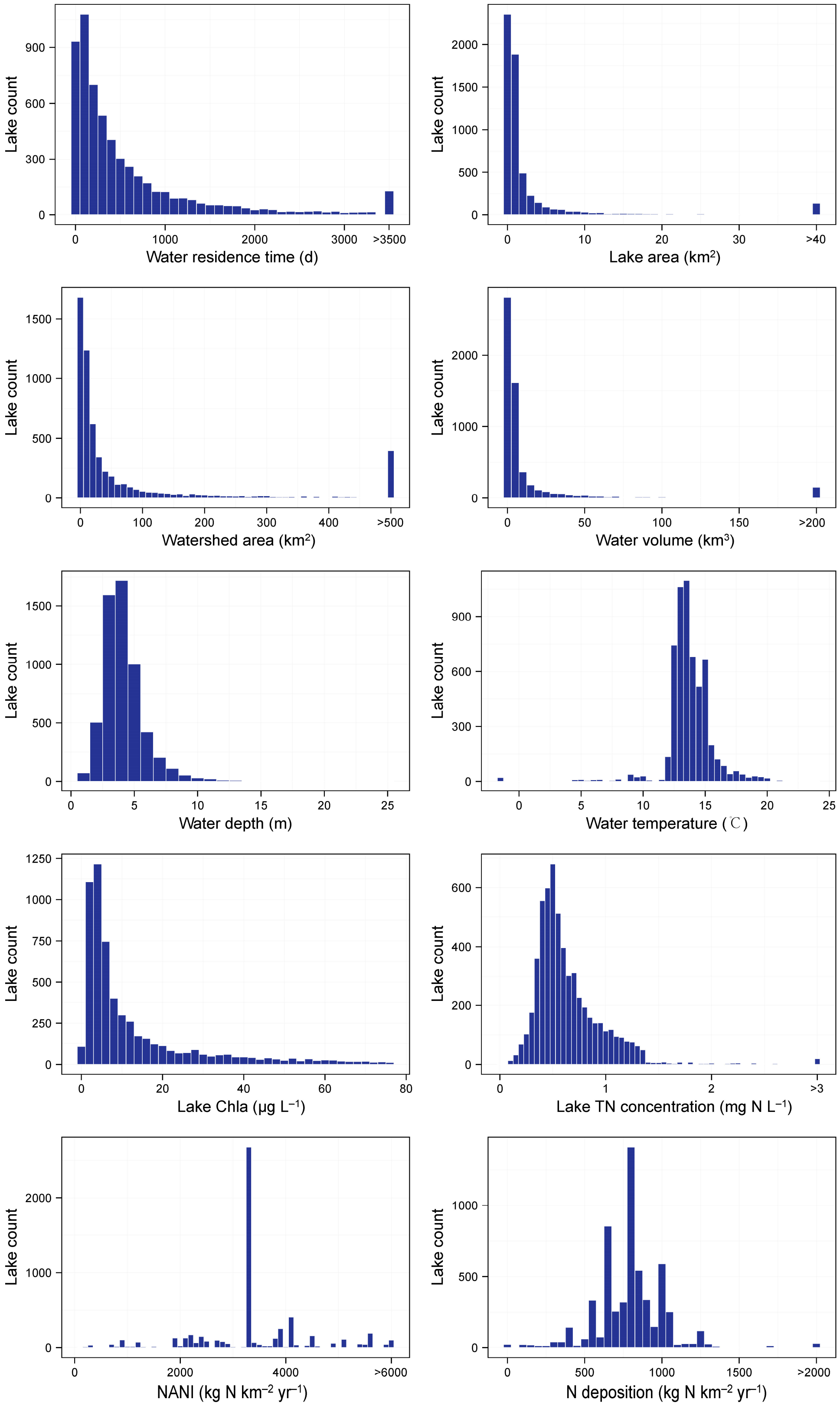

Supplementary Fig. 12 Distributions of lake properties for water quality improvement simulation in this study.

Supplementary References

1. Seitzinger, S. P. et al. Nitrogen retention in rivers: Model development and application to watersheds in the northeastern U.S.A. *Biogeochemistry* **57**, 199–237 (1902).
2. Penn, C. J., Warren, J. G. & Smith, S. Maximizing ammonium nitrogen removal from solution using different zeolites. *J. Environ. Qual.* **39**, 1478–1485 (2010).
3. Cheng, F. Y. & Basu, N. B. Biogeochemical hotspots: Role of small water bodies in landscape nutrient processing. *Water Resour. Res.* **53**, 5038–5056 (2017).
4. Li, X. et al. Sediment denitrification in waterways in a rice-paddy-dominated watershed in eastern China. *J. Soils Sediments* **13**, 783–792 (2013).
5. Zhao, Y. et al. Nitrogen removal capacity of the river network in a high nitrogen loading region. *Environ. Sci. Technol.* **49**, 1427–1435 (2015).
6. Piña-Ochoa, E. & Álvarez-Cobelas, M. Denitrification in aquatic environments: A cross-system analysis. *Biogeochemistry* **81**, 111–130 (2006).
7. David, M. B., Wall, L. G., Royer, T. V. & Tank, J. L. Denitrification and the nitrogen budget of a reservoir in an agricultural landscape. *Ecol. Appl.* **16**, 2177–2190 (2006).
8. Bruesewitz, D. A., Hamilton, D. P. & Schipper, L. A. Denitrification potential in lake sediment increases across a gradient of catchment agriculture. *Ecosystems* **14**, 341–352 (2011).
9. Jiang, X. et al. Salinity-linked denitrification potential in endorheic Lake Bosten (China) and its sensitivity to climate change. *Front. Microbiol.* **13**, 922546 (2022).
10. Müller, B., Meyer, J. S. & Gächter, R. Denitrification and nitrogen burial in Swiss Lakes. *Environ. Sci. Technol.* **56**, 2794–2802 (2022).

11. Liu, W., Jiang, X., Zhang, Q., Li, F. & Liu, G. Has submerged vegetation loss altered sediment denitrification, N_2O production, and denitrifying microbial communities in subtropical lakes? *Global Biogeochem. Cy.* **32**, 1195–1207 (2018).

12. Palacin-Lizarbe, C., Camarero, L., Hallin, S., Jones, C. M. & Catalan, J. Denitrification rates in lake sediments of mountains affected by high atmospheric nitrogen deposition. *Sci. Rep.* **10**, 3003 (2020).

13. Zhong, J. et al. The co-regulation of nitrate and temperature on denitrification at the sediment-water interface in the algae-dominated ecosystem of Lake Taihu, China. *J. Soils Sediments* **20**, 2277–2288 (2020).

14. She, D. et al. Limited N removal by denitrification in agricultural drainage ditches in the Taihu Lake region of China. *J. Soils Sediments* **18**, 1110–1119 (2018).

15. Yao, X., Zhang, L., Zhang, Y., Xu, H. & Jiang, X. Denitrification occurring on suspended sediment in a large, shallow, subtropical lake (Poyang Lake, China). *Environ. Pollut.* **219**, 501–511 (2016).

16. Zhang, L. et al. Influence of long-term inundation and nutrient addition on denitrification in sandy wetland sediments from Poyang Lake, a large shallow subtropical lake in China. *Environ. Pollut.* **219**, 440–449 (2016).

17. Chen, N., Wu, J., Chen, Z., Lu, T. & Wang, L. Spatial-temporal variation of dissolved N_2 and denitrification in an agricultural river network, southeast China. *Agric. Ecosyst. Environ.* **189**, 1–10 (2014).

18. Yan, X. et al. Dissolved organic carbon and dissolved oxygen determine the nitrogen removal rate constant in small water bodies of intensive agricultural region. *Agric. Ecosyst.*

Environ. **361**, 108822 (2024).

19. Filazzola, A. et al. A database of chlorophyll and water chemistry in freshwater lakes. *Sci. Data* **7**, 310 (2020).
20. European Union's Copernicus Land Monitoring Service information: Lake surface water temperature; https://land.copernicus.eu/global/products/lswt.
